# OPPORTUNITIES AND OUTCOMES OF CENTERING OLDER ADULTS IN PATIENT-ORIENTED AND COMMUNITY-ENGAGED RESEARCH

**DOI:** 10.1093/geroni/igac059.1449

**Published:** 2022-12-20

**Authors:** Carrie Leach, Carol Geary

## Abstract

Community engagement is critical for achieving context-sensitive and culturally appropriate communication, implementation and research approaches that can lead to better public and patient health outcomes. Though patient and community-engaged approaches are increasingly utilized in social research, the inclusion of older adults remains limited, as are methodologies that will help inform adult-centric programs and interventions. To advance the science of community engagement and implementation, this symposium brings together methodological innovations to help researchers shift from involving older adults as participants in research, toward collaboratively engaging and partnering with older adults in the design and conduct of research. The first presentation centers on place-based health disparities by starting with a statewide survey to identify communities that researchers could engage. The second highlights how the involvement of older adults led to the development of tactics for connecting with the hardly-reached, people over the age of 75, with health enhancing services and support. The third discusses a model for greater involvement of older adults to draw out lessons for implementation science in long-term care. The final presentation demonstrates an innovative approach to pilot new solutions with multidisciplinary and community partners in clinical settings. This CEnR and PPER interest group collaborative symposium concludes by imagining ways to integrate the diverse perspectives to advance the science of engaged research and implementation in community and clinical settings. This symposium represents diverse perspectives on involving community stakeholders meaningfully and equitably with research, to bend the later life health equity arc toward greater inclusion and justice.

